# Biomedical Application of Low Molecular Weight Heparin/Protamine Nano/Micro Particles as Cell- and Growth Factor-Carriers and Coating Matrix

**DOI:** 10.3390/ijms160511785

**Published:** 2015-05-22

**Authors:** Masayuki Ishihara, Satoko Kishimoto, Makoto Takikawa, Hidemi Hattori, Shingo Nakamura, Masafumi Shimizu

**Affiliations:** 1 Division of Biomedical Engineering Research Institute, National Defense Medical College, Saitama 359-8513, Japan; E-Mails: h2@ndmc.ac.jp (H.H.); snaka@ndmc.ac.jp (S.N.); 2 Research Support Center, Dokkyo Medical University, Tochigi 321-0293, Japan; E-Mail: skishi@dokkyomed.ac.jp; 3 Department of Medical Engineering, National Defense Medical College, Saitama 359-8513, Japan; E-Mail: drme003@ndmc.ac.jp; 4 Department of Surgery, Tokorozawa Meisei Hospital, Saitama 359-1145, Japan; E-Mail: masafumi@tokorozawameisei.or.jp

**Keywords:** adipose-derived stromal cells, bone marrow-derived mesenchymal stem cells, nano/micro particles, coating matrix, cell delivery systems

## Abstract

Low molecular weight heparin (LMWH)/protamine (P) nano/micro particles (N/MPs) (LMWH/P N/MPs) were applied as carriers for heparin-binding growth factors (GFs) and for adhesive cells including adipose-derived stromal cells (ADSCs) and bone marrow-derived mesenchymal stem cells (BMSCs). A mixture of LMWH and P yields a dispersion of N/MPs (100 nm–3 μm in diameter). LMWH/P N/MPs can be immobilized onto cell surfaces or extracellular matrix, control the release, activate GFs and protect various GFs. Furthermore, LMWH/P N/MPs can also bind to adhesive cell surfaces, inducing cells and LMWH/P N/MPs-aggregate formation. Those aggregates substantially promoted cellular viability, and induced vascularization and fibrous tissue formation *in vivo*. The LMWH/P N/MPs, in combination with ADSCs or BMSCs, are effective cell-carriers and are potential promising novel therapeutic agents for inducing vascularization and fibrous tissue formation in ischemic disease by transplantation of the ADSCs and LMWH/P N/MPs-aggregates. LMWH/P N/MPs can also bind to tissue culture plates and adsorb exogenous GFs or GFs from those cells. The LMWH/P N/MPs-coated matrix in the presence of GFs may provide novel biomaterials that can control cellular activity such as growth and differentiation. Furthermore, three-dimensional (3D) cultures of cells including ADSCs and BMSCs using plasma-medium gel with LMWH/P N/MPs exhibited efficient cell proliferation. Thus, LMWH/P N/MPs are an adequate carrier both for GFs and for stromal cells such as ADSCs and BMSCs, and are a functional coating matrix for their cultures.

## 1. Introduction

Drug repositioning is recognized as an effective strategy against the laborious and expensive *de novo* drug discovery as well as the difficult approval system of new drugs [1]. Recently low molecular weight heparin (Fragmin; LMWH) and protamine (P) have attracted attention as leading bionanomaterials in tissue engineering, cell-based therapy and regenerative medicine [1].

Basic protamine molecules complexed with acidic molecules such as heparin form complexes through ionic interactions [2]. We previously have reported the low molecular weight heparin/protamine nano/micro particles (LMWH/P N/MPs), which we originally prepared as polyelectrolyte complexes (PECs) [3,4]. LMWH/P N/MPs are specifically bound to fibroblast growth factor (FGF)-2 [3,4], hepatocyte growth factor (HGF) [5] and other heparin-binding growth factors (GFs) secreted from platelet-rich plasma (PRP) [6]. LMWH/P N/MPs can be retained onto cell surfaces and matrix in various tissues *in vivo* to control release, and can protect and activate GFs. Moreover, the GFs and LMWH/P N/MPs showed a substantial effect in inducing vascularization and fibrous tissue formations by stabilizing, activating and gradually releasing GFs from the GFs and LMWH/P N/MPs [6,7,8].

It was reported that LMWH/P N/MPs bind to various adhesive cell surfaces including ADSCs and BMSCs as well as tumor cells through specific interactions between LMWH/P N/MPs and cell surface heparin-binding proteins such as some integrins [9,10]. The interaction of the cells with LMWH/P N/MPs resulted in cells and LMWH/P N/MP-aggregate formation in a few hours. Those aggregates substantially promoted cellular viability *in vitro*. Injection of the aggregates induced vascularization and fibrous tissue formation *in vivo* [6,7]. Thus, LMWH/P N/MPs as cell carriers can enhance cell viability.

As a coating matrix, LMWH/P N/MPs were efficiently bound to tissue culture plates. With the ability of LMWH/P N/MPs to retain GFs, they could be very useful in cell culture. Human microvascular endothelial cells and human dermal fibroblast cells adhered well to LMWH/P N/MPs-coated suspension culture plates [10] and grew rapidly in low fetal bovine serum (FBS; 1%–2%) medium supplemented with FGF-2. This protocol could allow use of low autologous serum (1%–2%) in culturing BMSCs and ADSCs [7]. Furthermore, CD34+ hematopoietic progenitor cells (CD34+ HCs) derived from mouse bone marrow exhibited a higher proliferation on LMWH/P N/MPs-coated plates in hematopoietic progenitor growth medium (HPGM) supplemented with appropriate cytokines than those on uncoated plates [8]. Furthermore, ADSCs and BMSCs can also be grown efficiently in three-dimensional (3D) culture using low human plasma (HP) (3%)-DMEM gel containing LMWH/P N/MPs without animal serum [11,12]. Here, we describe LMWH/P N/MPs and their applications as GFs- and cell-carriers in tissue engineering, cell-based therapy and regenerative medicine, and as a coating matrix for cell cultures.

## 2. Biomedical Applications of Low Molecular Weight Heparin/Protamine Nano/Micro Particles (LMWH/P N/MPs)

### 2.1. Preparation and Function of Growth Factors (GFs) and LMWH/P N/MPs

Polyelectrolyte complexes (PECs) are generated by electrostatic interactions between oppositely charged polyelectrolytes, that is, LMWH and P. When this interaction occurs at non-equivalent ratios, nonstoichiometric PECs are produced, causing each PEC particle to carry an excess charge [13,14]. Proteins interact with both synthetic and natural PECs [15,16]. Heparin and LMWH specifically interact with functional proteins with high affinity including GFs, cytokines, extracellular matrix components and adhesion molecules [17,18,19]. Thus, heparin may be useful as a therapeutic agent in various pathological conditions that involve functional proteins, however, high-dose heparin cannot be used because of the excessive risk of bleeding [20]. In contrast, LMWH (approximately 5000 Da) has pharmacological and practical advantages compared with heparin. The lower protein binding activity of LMWH produces a low, stable and predictable anticoagulant response, thereby bypassing the need for laboratory monitoring of drug levels to adjust the dosage [20]. In addition, one or two subcutaneous injections per day are sufficient to maintain therapeutic concentrations because of its longer plasma half-life [20]. On the other hand, P (protamine), a purified mixture of proteins obtained from fish sperm, neutralizes heparin and LMWH by forming a stable complex that lacks anticoagulant activity [21]. Protamine is also employed in clinical use to reverse the anticoagulant activity of heparin following cardiopulmonary bypass as well as in cases of heparin-induced bleeding [22].

**Figure 1:**
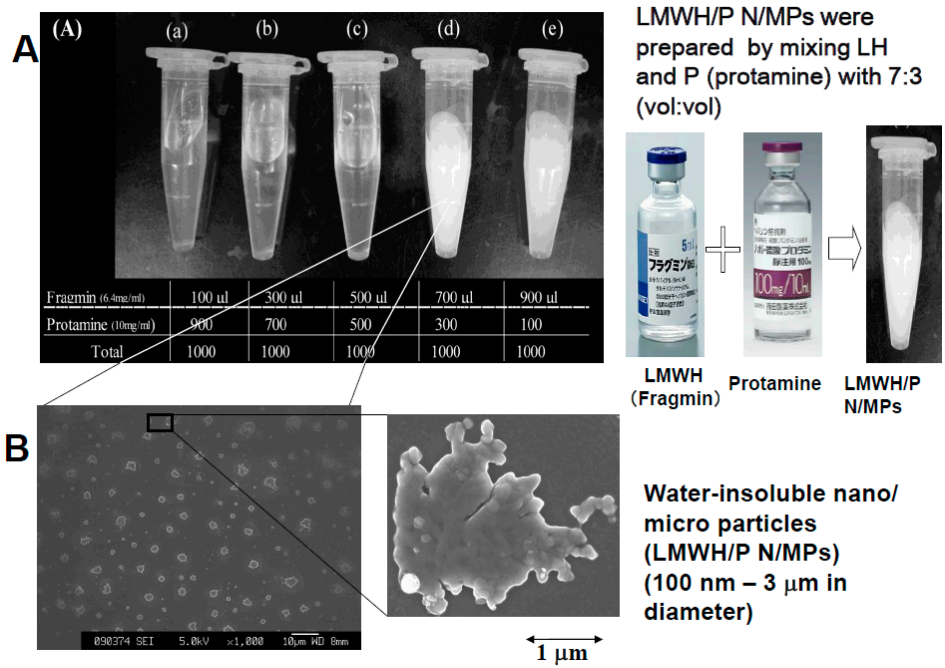
(**A**) Preparation and appearance of low molecular weight heparin/protamine nano/micro particles (LMWH/P N/MPs); (**B**) Electron microscopic appearance of generated LMWH/P N/MPs.

**Figure 2:**
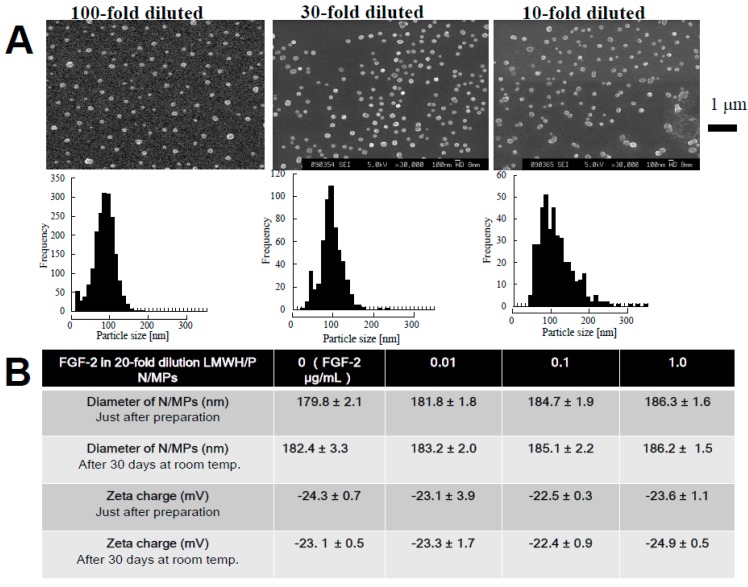
(**A**) Distribution of diameter of LMWH/P N/MPs; (**B**) Characterization of LMWH/P N/MPs and their stabilities. Stabilities of fibroblast growth factor (FGF)-2 and LMWH/P N/MPs were examined by measuring diameters of N/MPs and their zeta-electric charges.

We previously prepared water-insoluble N/MPs (100 nm–3 μm in diameter) by mixing LMWH (6.4 mg/mL) with P (10 mg/mL) at a ratio of 7:3 (*v*:*v*) (Figure 1). It was reported that LMWH/P N/MPs (100 nm–3 μm in diameter) have the ability to protect and to stimulate FGF-2 [3,4] and HGF activity [5]. GFs and LMWH/P N/MPs show a substantial effect to induce vascularization and fibrous tissue formation because of the gradual controlled release, protection and activation of GFs from GFs and LMWH/P N/MPs [3,4,5]. In another study, we used diluted LMWH (≤0.32 mg/mL) as an anion molecule and the diluted protamine (≤0.5 mg/mL) as a cation molecule to synthesize LMWH/P N/MPs (approximately 100 nm in diameter) (Figure 2A) [4]. LMWH/P N/MPs are unstable, and the particles irreversibly fuse and become larger (≥10 μm in diameter) during long-term storage (1 week or longer). Furthermore, after LMWH/P N/MPs are freeze-dried, cotton-like materials are produced, and these do not dissolve in water. The generated NPs could then be stabilized by adding dextran (*M*w: 178–217 kDa) and remained soluble after lyophilization of dialyzed LMWH/P N/MP solution or their concentrations by centrifugation [4]. The 20-fold diluted LMWH/P N/MPs (≈0.3 mg/mL) were stable for at least 30 days at room temperature, based on the changes of diameter of particles and their zeta-electric charges (Figure 2B) (data not published). In addition, FGF-2 and LMWH/P N/MPs [3,4] and HGF and LMWH/P N/MPs [5] were more mitogenic than each GF alone, because bound GF is more resistant to biodegradation and inactivation under physiological conditions.

### 2.2. Preparation of GFs in Platelet-Rich-Plasma (PRP) and LMWH/P N/MPs and Their Application

**Figure 3:**
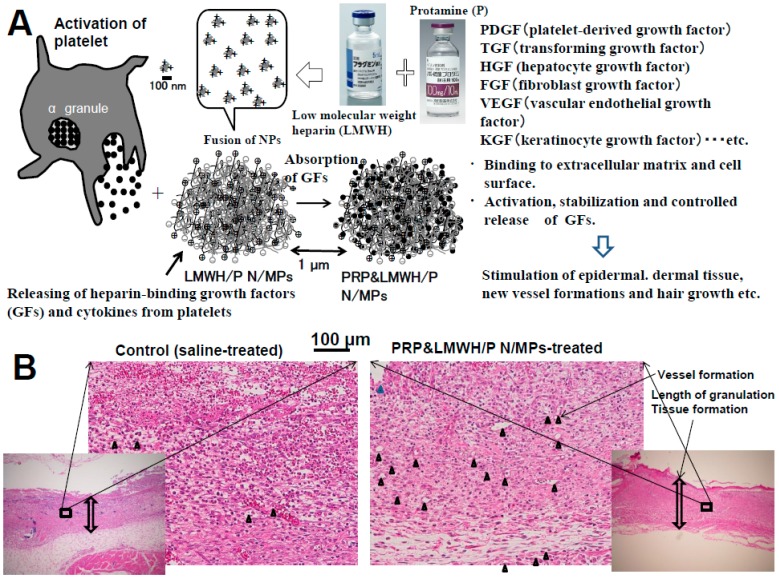
(**A**) Growth factors (GFs) from platelet-rich plasma (PRP) and LMWH/P N/MPs significantly induce neovascularization and granulation tissue formations. (**B**) Histological examinations of the base of wounds treated with saline (control), GFs from PRP and LMWH/P N/MPs-treated skin. Each photograph for wounds on day 7 was representative of eight rats. The vertical arrows show the length of formed granulation tissue, and black triangles show generated small blood vassels.

PRP contains a high concentration of platelets. Various GFs and other bioactive proteins secreted from the α-granules of platelets stimulate tissue repair and regeneration processes [6,23,24]. Platelets contain and secrete more than 20 GFs, including platelet-derived growth factors (PDGFs), FGFs, HGF, transforming growth factors (TGFs) and vascular endothelial growth factors (VEGFs), almost all of which are known to bind to heparin and heparin-like molecules (heparinoids) with high affinity (Figure 3A). Recent studies suggested that GFs from PRP not only influence the viability of transferred cells but may also play bioactive roles in the regulation of proliferation and differentiation in adipocyte precursor cells [25]. Clinical studies also suggested the efficacy and safety of PRP in the stimulation of repair and regeneration processes during hard- and soft-tissue formation [23,24].

The GFs from PRP are stably bound to LMWH/P N/MPs *in vivo*. The GFs from PRP adsorbed onto LMWH/P N/MPs may gradually be diffused and released upon biodegradation of LMWH/P N/MPs. Bound GFs to LMWH/P N/MPs are more resistant to biodegradation and inactivation under physiological conditions. Indeed, LMWH/P N/MPs could effectively protect each GF against heat inactivation and trypsin degradation [3,4,5]. The *in vivo* effects of GFs from PRP and LMWH/P N/MPs have also been demonstrated in neovascularization and formation of granulation tissue using enhanced filtration of inflammatory cells in nude mice [6]. When GFs from PRP and LMWH/P N/MPs were subcutaneously injected into the backs of mice, significantly higher neovascularization and granulation tissue formations with enhanced filtration of inflammatory cells were observed compared to the mouse group injected with GFs from PRP alone [6] (Figure 3B). Compared to either GFs in PRP alone or LMWH/P N/MPs alone, locally administered GFs from PRP and LMWH/P N/MPs augmented the wound bed and substantially increased viability of rat dorsal paired pedicle skin flaps [26]. The improved flap survival was noted if GFs from PRP and LMWH/P N/MPs was administered two days before the flap elevation [26]. GFs from PRP and LMWH/P N/MPs may thus represent a promising new biomaterial for improving skin flaps, particularly in the field of reconstructive surgery.

**Figure 4:**
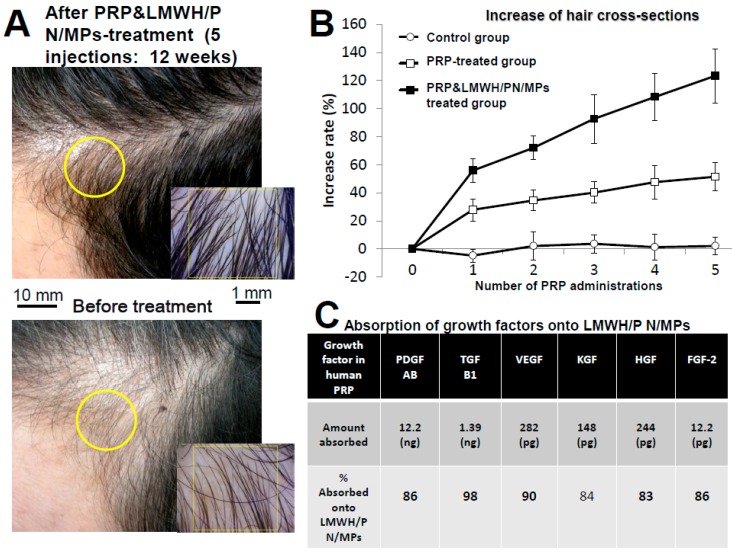
GFs from PRP and LMWH/P N/MPs treatment for ordinary baldness. (**A**) An appearances of hair before and after GFs from PRP and LMWH/P N/MPs-treatment (five injections for 12 weeks); (**B**) Hair cross-sections after administration of GFs from PRP and LMWH/P N/MPs (■), GFs from PRP alone (□), and saline alone (control: ○); (**C**) Binding of GFs onto LMWH/P N/MPs.

Clinical research (five injections, every two weeks) was performed using autologous GFs from PRP and LMWH/P N/MPs and GFs from PRP alone in 26 patients with ordinary baldness (including 10 women) (Figure 4A,B) [27]. Participants received a single subcutaneous injection of 3 mL of PRP and D/P MPs (67% PRP with 2 mg/mL of LMWH/P N/MPs in saline) or PRP (67% with saline) into the skin of the scalp using a 25-G needle. When 0.33 mL of LMWH/P N/MPs in saline (6 mg/mL) was added to 0.67 mL of activated PRP, incubated at room temperature for two hours, and centrifuged to separate the GFs from PRP and LMWH/P N/MPs, there were significantly fewer GFs in the supernatants, indicating that significant parts of those GFs were absorbed onto LMWH/P N/MPs (Figure 4C).

Hair regrowth and thickening following administration of GFs from PRP and LMWH/P N/MPs was observed in all patients compared with GFs from PRP alone and the control. GFs from PRP and LMWH/P N/MPs appeared to provide the most substantial change (Figure 4A,B) [27]. Because of the use of autologous materials, this method using GFs from PRP and LMWH/P N/MPs is simpler and cheaper, and has no side effects compared to conventional methods.

### 2.3. LMWH/P N/MPs as ADSCs- and BMSCs-Carriers

Tissue engineering or stem cell-based therapies require autologous multipotent stem cells such as ADSCs and BMSCs. Both cell types possess multipotentiality [28,29,30], as they differentiate in culture or after implantation *in vivo* into osteoblasts [31,32], chondrocytes [33,34], adipocytes [35], myotubes [36,37], and neuronal cells [38,39]. Several reports indicate that the transplantation of ADSC-cultured constructs into athymic mice stimulates angiogenesis, wound repair, and re-epithelialization significantly better, when compared with fibroblast-cultured constructs [9,40,41]. Thus, the use of ADSCs and BMSCs is a promising tissue engineering strategy.

However, the therapeutic efficacy of stem cell therapy is often limited by poor performance in engraftment—probably because of the rapid disappearance of grafted cells from the injection site [42,43]. Therefore, for applications of ADSCs and BMSCs, adequate cell-carriers seem to be necessary. Low molecular weight heparin (LMWH) and protamine (P) have been repurposed as a cell-carrier in tissue engineering, cell-based therapy and regenerative medicine [7,9].

It was reported that LMWH/P N/MPs bind to cell surfaces of ADSCs and BMSCs through specific interactions between LMWH/P N/MPs and cell surface heparin-binding proteins such as some integrins. The interaction of the cells with LMWH/P N/MPs resulted in cells and LMWH/P N/MPs-aggregate formation. Those aggregates substantially promoted cellular viability *in vitro*. Injection of the aggregates induced vascularization and fibrous tissue formation *in vivo* [9].

**Figure 5:**
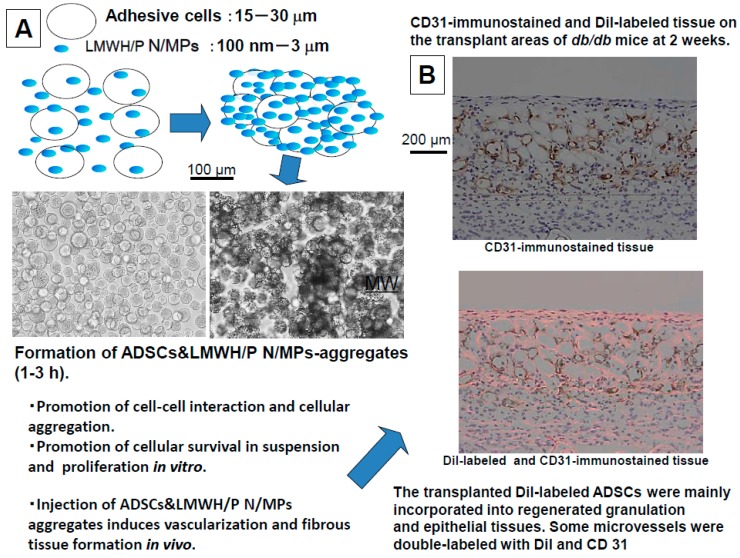
Mechanism of formation of cells and LMWH/P N/MPs-aggregates and *in vivo* activities. (**A**) Formation of ADSCs&LMWH/P N/MPs; (**B**) Immunostained and Dil-labeled tissue.

LMWH/P N/MPs as cell-carriers can substantially enhance cell viability, including human microvascular endothelial cells (hMVECs), human dermal fibroblasts (hDFCs) and ADSCs [10]. In particular, ADSCs have the potential to differentiate into skin, bone, cartilage, fat, myocardium, skeletal muscle and neurons [30,32]. ADSCs can easily be harvested with lower donor site morbidity compared with other pluripotent stem cell sources. Furthermore, ADSCs can easily attach and proliferate in culture, and therefore, are available on a large scale even for autologous grafting in small animals such as rodents [7,10,11]. However, using ADSCs for therapeutic angiogenesis and vasculogenesis requires cell-carriers to act as injectable vehicles necessary for transplantation of ADSCs. It was observed that LMWH/P N/MPs could bind to the surface of the cells as mentioned above. The interaction of these cells with LMWH/P N/MPs induced ADSCs and LMWH/P N/MPs-aggregate formation, and substantially maintained cell viability during at least three days in cell suspensions compared to that of control (Figure 5A). The ADSCs and LMWH/P N/MPs-aggregates adhered and grew on suspension culture plates, and the aggregates similarly grew on type I collagen-coated plates. Furthermore, cultured ADSCs secreted a significant amounts of angiogenic GFs such as FGF-2, HGF, PDGF and VEGF. These secreted GFs could have been retained within the ADSCs and LMWH/P N/MPs-aggregates. When the ADSC and LMWH/P N/MPs-aggregates were subcutaneously injected into the backs of nude mice, a significant increase in neovascularization and fibrous tissue formation was observed near the injected site from three days to two weeks [6,9]. By using fluorescent (DiI) labeling of ADSCs from *db*/*db* mice and autologous transplantation, we investigated the fates of autologous ADSCs seeded into healing-impaired wounds, the differentiation of autologous ADSCs, and the interactions between seeded autologous ADSCs and their environment. The results suggested that the majority of seeded autologous ADSCs were incorporated into the regenerated granulation and epithelial tissues [44]. In addition, some of the seeded ADSCs may incorporate into blood vessels as indicated by CD31-immunostaining (Figure 5B). Taken together, these data indicate that ADSCs and LMWH/P N/MPs-aggregates are useful biomaterials for angiogenesis and wound repair cellular therapy. Thus, interaction of adhesive cells with LMWH/P N/MPs induced ADSCs and LMWH/P N/MPs-aggregate formation, and substantially promoted cell viability in cell suspensions *in vitro*, and injections of the aggregates significantly enhanced vascularization and fibrous tissue formations *in vivo* [6,9]. However, ADSCs and LMWH/P N/MPs-aggregates could not be injected intravenously because of serious risk of thrombosis.

The LMWH/P N/MPs also bind to the surface of tumor cells (e.g., 3LL, B16 and Huh7), promote cell-to-cell interaction, and increase the aggregation of the tumor cells with the LMWH/P N/MPs. Those tumor cells and LMWH/P N/MPs-aggregates substantially promote cell survival and proliferation of the tumor cells *in vitro* as well as reliably induce tumor formation and rapid tumor growth *in vivo* [44]. Those results indicated that LMWH/P N/MPs constitute an effective biomaterial that functions as a tumor cell carrier *in vivo*. The application of LMWH/P N/MPs as a tumor cell carrier offers a more reliable model in both allograft and xenograft transplantation for cancer research [45,46].

### 2.4. Cell Cultures with Low Serum Using LMWH/P N/MPs-Coated Plates

**Figure 6:**
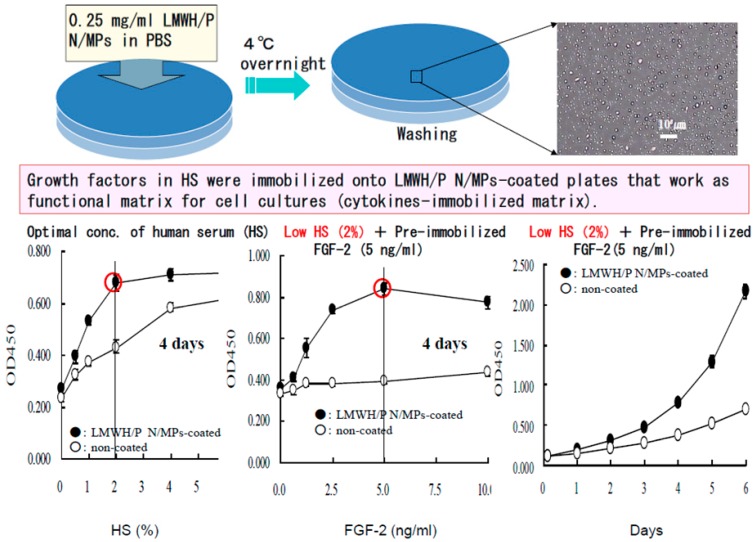
Expansion of adipose-derived stromal cells (ADSCs) with LMWH/P NPs-coated plates and human serum (HS). ADSCs effectively expanded with low HS (2%) and FGF-2 (5 ng/mL) instead of high FBS (10%) on LMWH/P N/MP-coated plates.

Heparinoids bind various heparin-binding growth factors (GFs) and cytokines including FGFs, HGF, VEGF, heparin-binding epidermal growth factor (HBEGF), PDGF, TGF-β, granulocyte/macrophage-colony stimulating factor (GM-CSF), interleukins (*i.e.*, IL-1, IL-2, IL-3, IL-4, IL-6, IL-7 and IL-8), interferon γ and macrophage inflammatory protein-1 [17,18,19]. We have already reported that FGF-1, HGF, HBEGF, TGF-β, human stem cell factor (SCF), recombinant human thrombopoietin (Tpo) and recombinant human Flt-3-ligand (Flt-3) could be efficiently immobilized on the LMWH/P N/MPs-coated plates [8,10]. Thus, LMWH/P N/MPs-coating matrix provides an excellent biomaterial to immobilize and retain GFs and cytokines for superior growth of various types of cells with low (1%–2%) serum (either fetal bovine serum (FBS) or human serum (HS)) medium. The LMWH/P N/MPs generate a stable paste-like coating through complete drying. It is probable that polypeptides, such as FGF-2, IL-3 and GM-CSF, once bound to the LMWH/P N/MPs-coated plates, are gradually released from the coated surface *in vitro* with a half-life of four to six days [8,10]. Furthermore, LMWH/P N/MPs-coating could optimally stimulate growth of hMVECs and hDFCs in low HS (1%–2%)-DMEM with FGF-2 and growth of hematopoietic cell line (TF-1) with IL-3 and GM-CSF [10,11]. Furthermore, ADSCs and BMSCs proliferated in DMEM using low-concentration serum (2% HS or FBS) on FGF-2 immobilized LMWH/P N/MPs-coated plates (Figure 6) [7].

Cell-based therapies such as tissue engineering will benefit from a source of autologous multipotent stem cells including BMSCs and ADSCs. There are two stem cell lineages in bone marrow cell populations, *i.e.*, hematopoietic cells (HCs) and BMSCs. The BMSCs and ADSCs are multipotential, indicating that in culture [1,2,3] or after *in vivo* implantation these cells can differentiate into a variety of cell types including osteoblasts, chondrocytes and adipocytes *etc.*

Most protocols for the *in vitro* expansion of BMSCs or ADSCs include high concentrations (10%–20%) of animal serum such as fetal bovine serum (FBS) as a nutritional supplement. Some methods for cell cultures involve multiple doses of FBS, which raises concerns over possible contamination as well as immunological reactions caused by medium-derived FBS proteins or sialic acid derivatives [47,48]. Patients may experience problems when undergoing autologous cell-based therapies if a serum other than an autologous serum is used during the culturing of the cells. On the other hand, it would be difficult to obtain large amounts of autologous serum from the patient for large-scale autologous cell culture [7,49]. It should be noted that the cell growth of cultured BMSCs and ADSCs on LMWH/P N/MPs-coated plates in low FBS (1%–2%) medium with FGF-2 was significantly stimulated, and similar stimulation was observed in those cultured cells on LMWH/P N/MPs-coated plates with FGF-2 and 1%–2% human serum (HS) prepared from adult bloods instead of FBS. Thus, LMWH/P N/MPs may serve as a functional matrix for cultures of BMSCs and ADSCs. The safe and effective expansions of BMSCs and ADSCs represent a promising option for tissue engineering- and cell therapy-strategies.

### 2.5. Proliferation of CD34+ Hematopoietic Progenitor Cells (CD34+ HCs) on LMWH/P N/MPs-Coated Plates

Hematopoietic progenitor cells from bone marrow (HCs) proliferate and mature in semi-solid media when stimulated by exogenous hematopoietic cell growth factors (HCGFs) such as SCF, Tpo, Flt-3, IL-3 and GM-CSF [50,51,52]. HCs also expanded in association with bone marrow-derived stromal cells [7,48,53,54], although biologically active amounts of HCGFs cannot be detected in stromal culture supernatants [54]. It is possible that HCGFs are synthesized by the stromal cells but remain bound to the stromal cells and their extracellular matrix. In fact, it was demonstrated that both natural and recombinant HCGFs could be adsorbed by heparan sulfate, which is the major sulfated glycosaminoglycan of bone marrow stroma [51,52,53,54]. However, it was reported that serum-free media require large amounts of SCF, Tpo and Flt-3 to proliferate CD34+ HCs [8,49,54,55,56]. Although such media are commercially available (HPGM, Lonza Japan Corp. Tokyo, Japan), they are prohibitively expensive. In our previous study, we demonstrated that recombinant HCGFs were immobilized onto LMWH/P N/MPs-coated plates, and the immobilized cytokines were gradually released into the medium. Furthermore, these cytokines, once bound, can be presented in the biologically active form to HCs [8,50]. Furthermore, the much lower concentrations of the cytokines than those recommended by the manufacturer (Lonza Japan Corp.) were required for maximal expansion of CD34+ HCs on the LMWH/P N/MPs-coated plates [8,50]. Those findings may have important implications for the use of heparinoid as an artificial matrix for *ex vivo* expansion of CD34+ HCs with adequate cytokines. The LMWH/P N/MPs-coating matrix in the presence of much lower concentrations of SCF, Tpo and Flt-3 is a convenient and safe material for superior expansion of CD34+ HCs using HPGM without any animal serum [8,50].

### 2.6. Three-Dimensional Expansion of ADSCs and BMSCs Using Plasma-Medium Gel with LMWH/P N/MPs

**Figure 7:**
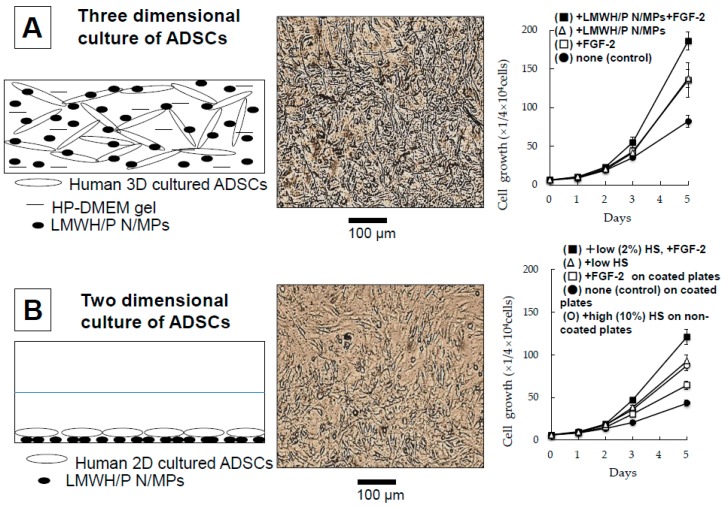
Three dimensional culture of ADSCs with HP-DMEM gel and LMWH/P NPs. (**A**) Three dimensional culture of ADSCs; (**B**) Two dimensional culture of ADSCs.

Plasma-medium gel provides an extracellular matrix for the culture of different cell types by forming a massive capsule with semi-permeable properties that allows the diffusion of medium components into cells and elimination of waste. Both exogenous and endogenous GFs efficiently bind to LMWH/P N/MPs, with their activities remaining stable. Human ADSCs and BMSCs can be grown in two-dimensional (2D) culture using low human serum (HS) (1%–2%) and Dulbecco’s modified Eagle’s medium (DMEM) with sufficient cytokines on LMWH/P N/MPs-coated tissue culture plates [7]. Furthermore, ADSCs and BMSCs can also be grown efficiently in three-dimensional (3D) culture using low human plasma (HP) (2%)-DMEM gel containing 0.1 mg/mL LMWH/P N/MPs without animal serum [11,12]. Furthermore, the phenotypes of both cell types were positive for CD44, CD90 and CD105 (>80%) and negative for CD34 and CD45 (<1%) [12]. Thus, those cells were well maintained in 2D and 3D cultures after seven days. The 3D-proliferated ADSCs and BMSCs maintained their multipotent differentiation capacity, that is, they were able to differentiate into adipocytes and osteoblasts [12]. Those results demonstrated the superior proliferation of both cell types using the 3D culture system in low-concentration HP-DMEM gel with LMWH/P N/MPs and FGF-2. The 3D culture system for cultures of ADSCs or BMSCs is composed of low-concentration HP-medium gel with LMWH/P N/MPs and FGF-2. ADSCs grew better in 3D culture (Figure 7A) than in 2D culture (Figure 7B). Furthermore, the presented 3D culture methods required no animal serum, because the low concentration (2%) of autologous plasma was sufficient [12].

### 2.7. Transplantation of Inbred Rat Adipose-Derived Stromal Cells (IR-ADSCs) Using Plasma Gel with LMWH/P N/MPs/FGF-2

Various biomaterials have been used as cell carriers during cell implantations [57,58]. Those materials should fulfill several preconditions such as biocompatibility, biodegradability, low cytotoxicity and high affinity for the biological surface [57,58]. The biomaterial tested in this study, inbred rat plasma (IRP)-DMEM gel with LMWH/P N/MPs, can carry many IR-ADSCs and also act as a cell carrier in which the cells can grow. It has been reported that many LMWH/P N/MPs can bind to surfaces of ADSCs, and the interaction of ADSCs with LMWH/P N/MPs induces ADSCs and LMWH/P N/MP-aggregate formation, and substantially maintains cell viability for at least three days in suspension-culture conditions [9]. In contrast, it appears that the interaction of IR-ADSCs in IRP-DMEM gel as a 3D matrix stimulates the growth of IR-ADSCs and generates a 3D network [11,12]. Furthermore, the growth factors secreted from IR-ADSCs as well as the growth factors derived from the IRP may be retained within the IRP-DMEM gel with LMWH/P N/MPs [58].

**Figure 8:**
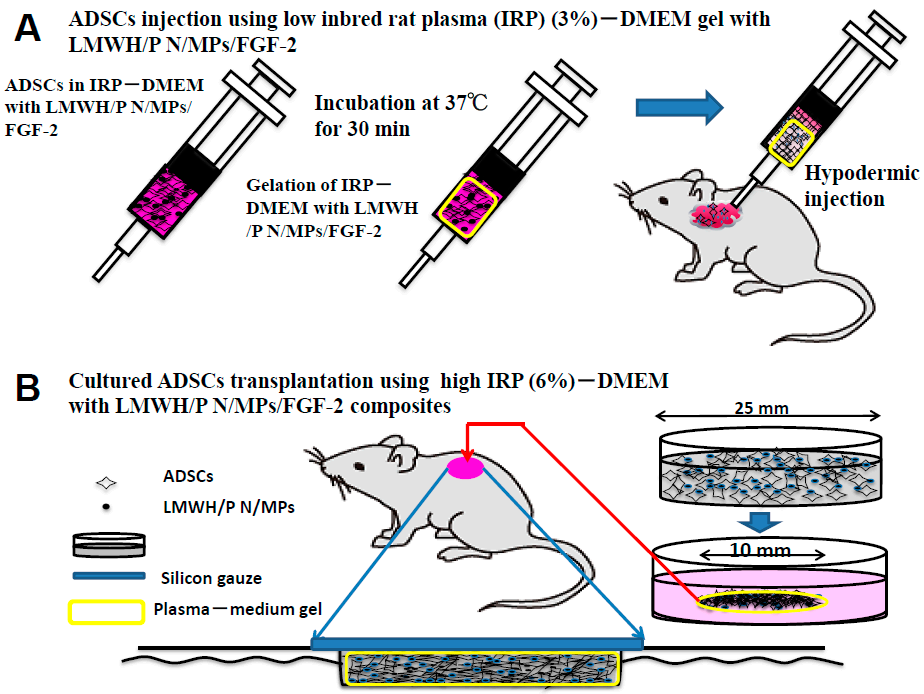
Two methods (**A**,**B**) for ADSCs transplantations using IRP-DMEM gel with LMWH/P N/MPs/GF.

We have previously applied 3D-cultured IR-ADSCs derived from inbred male Fisher 344 rats using injectable low IRP (3%)-DMEM gel with LMWH/P N/MPs for cell transplantation (Figure 8A) [58]. Figure 8B showed the application of IR-ADSCs using IRP (6%)-DMEM gel with LMWH/P N/MPs/FGF-2 to full thickness skin excisions as healing-impaired wounds on the backs of STZ-induced diabetic rats [59]. The wound closures treated with IR-ADSCs using IRP-DMEM gel with LMWH/P N/MPs/FGF-2 were significantly enhanced on post-wounding [59]. The histological examination of wounds treated with IR-ADSCs using IRP-DMEM gel with LMWH/P N/MPs/FGF-2 demonstrated significantly advanced epithelialization, capillary formation and granulation tissue formation. Those results indicate that a portion of the transplanted IR-ADSCs using IRP-DMEM gel with LMWH/P N/MPs/FGF-2 had been taken up by the granulation tissues to promote wound healing [59]. Thus, those studies suggest that IR-ADSCs using IRP-DMEM gel with LMWH/P N/MPs is effective for repairing healing-impaired wounds such as those occurring in diabetes.

## 3. Conclusions

Low molecular heparin (LMWH) and protamine (P) have been clinically utilized to control circulating heparin. LMWH and P have been recently repurposed in tissue engineering, cell-based therapy and regenerative medicine as LMWH/P N/MPs [1]. Firstly, LMWH/P N/MPs can function as carrier for GFs including FGF-2, HGF and GFs from PRP, which are retained locally, and control the release and protect them. GFs and LMWH/P N/MPs could induce vascularization and fibrous tissue formation *in vivo*. Secondly, cells and LMWH/P N/MPs-aggregates formation substantially promoted cell viability *in vitro*, and the cells and LMWH/P N/MPs-aggregates induced vascularization and fibrous tissue formation *in vivo* [9]. The LMWH/P N/MPs may be a promising new drug for cell-based therapy as superior cell-carriers.

The methods presented allowed superior proliferation and differentiation of ADSCs and BMSCs by utilizing FGF-2 on LMWH/P N/MPs-coated plates in low concentration human serum (1%–2%) medium. The proliferated cells maintained their potential to differentiate into adipocytes and osteoblasts [7]. Furthermore, the LMWH/P N/MPs-coating matrix in the presence of lower concentrations of SCF, Tpo and Flt-3 were effective materials for the stable expansion of CD34+ HCs using HPGM without any animal serum [8]. Those results suggest a sufficient cell source, particularly for the preparation of large amounts of ADSCs, BMSCs or CD34+ HCs required for cell-based therapies in several clinical fields, including angiogenesis, dermatological and regenerative medicines.

LMWH, P, GFs from autologous PRP and several recombinant GFs and cytokines are already in clinical use. Since autologous ADSCs, BMSCs or CD34+ HCs are available, the clinical safety of LMWH/P N/MPs as cell-carriers is promising. Furthermore, ADSCs, BMSCs or CD34+ HCs can be efficiently expanded as cell sources for regenerative medicines with the use of LMWH/P N/MPs-coated plates as a functional matrix without the need for feeder cells or animal serum.

The superior proliferation of both ADSCs and BMSCs using the 3D culture system in low-concentration HP-DMEM gel with LMWH/P N/MPs and FGF-2 was also demonstrated. The 3D culture system for cultures of ADSCs or BMSCs is composed of low-concentration HP-medium gel with LMWH/P N/MPs and FGF-2. Those cells grew better in 3D culture than in 2D culture. The 3D culture methods presented required no animal serum, because the low concentration (3%) of autologous plasma was sufficient. Furthermore, we have successfully applied 3D-cultured IR-ADSCs using injectable low IRP (3%)-DMEM gel with LMWH/P N/MPs to *in vivo* cell transplantation treatment for repairing healing-impaired wounds such as those occurring in diabetes [58,59].
